# Self-Organized Nanoparticles of Random and Block Copolymers of Sodium 2-(Acrylamido)-2-methyl-1-propanesulfonate and Sodium 11-(Acrylamido)undecanoate as Safe and Effective Zika Virus Inhibitors

**DOI:** 10.3390/pharmaceutics14020309

**Published:** 2022-01-27

**Authors:** Pawel Botwina, Magdalena Obłoza, Maria Zatorska-Płachta, Kamil Kamiński, Masanobu Mizusaki, Shin-Ichi Yusa, Krzysztof Szczubiałka, Krzysztof Pyrc, Maria Nowakowska

**Affiliations:** 1 Microbiology Department, Faculty of Biochemistry, Biophysics and Biotechnology, Jagiellonian University, 30-387 Krakow, Poland; pawel.botwina@doctoral.uj.edu.pl; 2 Virogenetics Laboratory of Virology, Malopolska Centre of Biotechnology, Jagiellonian University, 30-387 Krakow, Poland; 3 Department of Physical Chemistry, Faculty of Chemistry, Jagiellonian University, 30-387 Krakow, Poland; m.obloza@uj.edu.pl (M.O.); maria.zatorska@doctoral.uj.edu.pl (M.Z.-P.); kaminski@chemia.uj.edu.pl (K.K.); k.szczubialka@uj.edu.pl (K.S.); 4 Department of Applied Chemistry, Graduate School of Engineering, University of Hyogo, 2167 Shosha, Himeji 671-2280, Japan; mizusaki.masanobu@sharp.co.jp (M.M.); yusa@eng.u-hyogo.ac.jp (S.-I.Y.)

**Keywords:** Zika virus, sodium 2-(acrylamido)-2-methyl-1-propanesulfonate, sodium 11-(acrylamido)undecanoate, amphiphilic copolymers

## Abstract

A series of anionic homopolymers, poly(sodium 2-(acrylamido)-2-methyl-1-propanesulfonate) (PAMPS) and amphiphilic copolymers of AMPS and sodium 11-(acrylamido)undecanoate (AaU), both block (PAMPS_75_-b-PAaU_n_), and random (P(AMPS_m_-co-AaU_n_)), were synthesized and their antiviral activity against Zika virus (ZIKV) was evaluated. Interestingly, while the homopolymers showed limited antiviral activity, the copolymers are very efficient antivirals. This observation was explained considering that under the conditions relevant to the biological experiments (pH 7.4 PBS buffer) the macromolecules of these copolymers exist as negatively charged (zeta potential about −25 mV) nanoparticles (4–12 nm) due to their self-organization. They inhibit the ZIKV replication cycle by binding to the cell surface and thereby blocking virus attachment to host cells. Considering good solubility in aqueous media, low toxicity, and high selectivity index (SI) of the PAMPS-b-PAaU copolymers, they can be considered promising agents against ZIKV infections.

## 1. Introduction

Zika virus (ZIKV) is a positively stranded RNA virus that belongs to the *Flaviviridae* family. The family encompasses a number of viruses, including dengue virus (DENV), West Nile virus (WNV), and yellow fever virus (YFV) [1]. Zika virus was first isolated in the forests of Uganda from a sample collected from rhesus monkey in 1947 [2]. The transmission cycle of ZIKV used to be primarily sylvatic and was limited to primates and mosquitos of *Aedes* species [3]. The first case of Zika virus-associated disease in humans was recorded in 1954 during an outbreak in Nigeria [4], and until 2007, ZIKV was associated with sporadic infections in humans.

In 2007, the first outbreak of ZIKV was reported on Yap islands and the Federal States of Micronesia. Up to 73% of the population of the islands above the age of three were infected with ZIKV, and ~20% of these exhibited clinical symptoms [5]. The next ZIKV outbreak in humans was identified on French Polynesia in 2013–2014, with an estimated 28,000 cases (11.5% of the Polynesian population) [6,7]. The virus reached the Western Hemisphere in Brazil in 2013 and rapidly spread throughout Central and Southern America, including the United States [8,9,10]. More than 800,000 documented and suspected human cases of ZIKV were reported in the Americas from 2015 to 2018, along with potentially millions of undiagnosed infections [11]. Most infections remain asymptomatic or manifest with mild symptoms, including fever, rash, arthralgia, or conjunctivitis. However, ZIKV is neurotropic, and it can cause severe neurological complications, including the Guillain-Barré syndrome (GBS) [12] and congenital Zika syndrome (CZS) [13] in fetuses. The absolute risk of adverse birth outcomes (i.e., miscarriage, stillbirth, premature birth, and CZS) has been estimated to range between 7 and 46% [8,14,15,16,17]. Currently, there are no specific antiviral drugs or vaccines available to treat or prevent ZIKV infections.

The strategies using polymeric macromolecules as antivirals have been widely studied. Initially, natural polymers were used as backbones (e.g., polysaccharides), but soon the research expanded to also utilize synthetic polymers [18,19,20,21]. High efficacy, ease of modification and low toxicity compared to small molecule drugs make them attractive candidates for antiviral drugs. Moreover, polymers are often effective in inhibiting more than one type of virus, which can be useful in countering diseases with diverse etiological factors, e.g., the common cold. Antiviral polymers typically block virus attachment or entry into cells by creating a protective macromolecular barrier on the cell surface or interacting with the virions themselves [22,23,24].

Moreover, polymers exhibit a phenomenon called “polyvalency” when multiple repeating units of the polymer can bind to multiple complementary cell receptors or viral proteins simultaneously [19,25]. As multiple individual ligand–receptor interactions act synergistically, polyvalent interactions are typically much stronger than monovalent binding. That can be affected by selecting the polymeric structures adopting spherical conformation in aqueous media and exposing the functional binding groups. Such a strategy was applied in the current work. A series of amphiphilic copolymers of sodium 2-acrylamido-2-methylpropanesulfonate (AMPS) and sodium 11-acrylamido undecanoate (AaU), both block (PAMPS_75_-b-PAaU_n_) and random (P(AMPS_50_-co-AaU_50_), undergoing self-organization in aqueous media with the formation of spherical, highly charged core-shell micelles were synthesized and tested for their ability to hamper ZIKV replication cycle in vitro [26]. Their antiviral activity was compared with poly(2-acrylamido-2-methyl-1-propanesulfonic acid) (PAMPS) polyanions, including one with the same number of charged sulfonic units. It was observed that while PAMPS-PAaU copolymers (block and random) exhibited strong antiviral efficacy against ZIKV, the ionic homopolymers PAMPS showed relatively limited activity. The copolymers efficiently inhibit the ZIKV-caused cytopathic effect (CPE) and virus yield in cell culture supernatants at very low concentrations (IC_50_ < 1 µg/mL). Additionally, the tested copolymers show low toxicity with a CC_50_ above 2000 µg/mL. The mechanistic studies indicated that the polymers studied blocked virus attachment to the host cells, preventing its entry. That process is expected to be driven by electrostatic interactions between the negatively charged sulfonate units present in polymers studied and positively charged glycoproteins located at the cell surface. Interestingly, however, the mechanism of antiZIKV activity of copolymers PAMPS and PAaUn differs considerably from that identified by us for poly(sodium 4-styrenesulfonate) (PSSNa). We demonstrated recently that poly(sodium 4-styrenesulfonate) (PSSNa) inhibits ZIKV replication in vitro both in animal and human cells mostly by binding to the ZIKV particle, thus blocking its attachment to the host cells [24]. The process is efficient when the polymer of high molecular weight is applied. The comparison of the antiviral efficiency of PAMPS homopolymer and copolymers having the same numbers of PAMPS units indicates that this is the self-organization of the polymer chains that plays an essential role in that process. This is because PAMPS homopolymers, being strong polyelectrolytes, adopt rather extended conformation in water (with hydrodynamic radiuses, *R*_h_, and zeta potentials, ζ, 2–4 nm and −20 mV, respectively), while PAMPS-PAaU copolymers in aqueous media form negatively charged nanosized micellar particles with *R*_h_ and ζ of 5–12 nm and −25 mV, respectively. They can compete with virus particle interactions with cell receptors. Such mechanism of action was recently demonstrated for nanoballs of tridecafullerenes appended with up to 360 1,2-mannobioside units. These particles inhibited entry of ZIKV and Dengue viruses (DENV) to the host cell by blocking the intercellular adhesion molecule-3-grabbing nonintegrin (DC-SIGN) [27,28]. While they are effective as antivirals, the synthesis is demanding. The macromolecular systems proposed in the current paper can be easily prepared using well-mastered reversible addition-fragmentation chain transfer (RAFT) radical polymerization. Their structure, composition, molecular mass and resulting biological properties can be adjusted accordingly. Thus, the homopolymers or copolymers best suited to counteract viral infection can be designed and synthesized.

## 2. Materials and Methods

**Figure 1:**
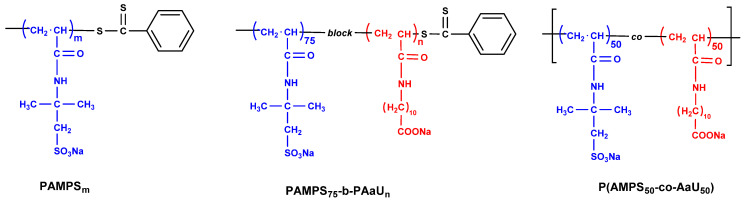
Chemical structures of PAMPS homopolymer, diblock PAMPS_75_-b-PAaU_n_ copolymers and random P(AMPS_50_-co-AaU_50_) copolymer.

PAMPS homopolymers and diblock copolymers (Figure 1) composed of poly(sodium 2-acrylamido-2-methyl-1-propanesulfonate) (PAMPS) and poly(sodium 11-acrylamido undecanoate) (PAaU) were synthesized via reversible addition-fragmentation chain transfer (RAFT) radical polymerization following the procedure described earlier [26].

A typical synthesis of PAMPS_75_-b-PAaU_n_ is given in Supplementary Materials. Random copolymer P(AMPS_50_-co-AaU_50_) was prepared via free radical polymerization using the procedure described earlier (for details see Supplementary Materials) [29]. The synthesis and characterization of fluorescently labeled copolymer PAMPS_75_-b-PAaU_28_-b-F is described in Supplementary Materials.

Size Exclusive Chromatography (SEC) analysis was performed at 40 °C with a reflective index (RI) detector equipped with a Shodex 7.0 µm bead size GF-7F HQ column (exclusion limit ~107) using an appropriate eluent at a flow rate of 0.6 mL/min. Mn, weight-average molecular weight (Mw), and Mw/Mn of the polymer were calibrated with standard poly(sodium styrenesulfonate) samples of 11 different molecular weights ranging from 1.37 × 10^3^ to 2.61 × 10^6^.

UV-Vis spectra were collected at RT in 1 cm quartz cuvettes using single beam photodiode array with Hewlett-Packard 8452A spectrophotometer (Palo Alto, CA, USA) with a resolution of 2 nm in the range of 190–820 nm.

The ^1^H NMR spectra were obtained with a Bruker DRX-500 spectrometer (Billerica, MA, USA) operating at 500 MHz in D2O and Bruker Avance II 600 MHz in DMSO. Chemical shifts were determined by using 3-(trimethylsilyl)propionic-2,2,3,3-d4 acid as an internal reference. The 1H NMR spin-spin relaxation times (T2) were determined by the Carr–Purcell–Meiboom–Gill (CPMG) method [30]. A 90° pulse of 13.85 µs was calibrated and used for the measurement. Peak intensities at 12 different numbers of 180° pulse were measured.

Nano ZS instrument (Malvern Instrument, Worcestershire, UK) was applied for zeta potential (ZP) and dynamic light scattering (DLS) measurements. The light scattering angle was equal to 173°. ZP measurements were performed using the laser Doppler velocimetry technique. Folded capillary cells were utilized in all measurements.

### 2.1. Cells and Virus

Vero cell line (*Cercopithecus aethiops* kidney epithelial, ATCC CCL-81) (ATCC, Manassas, VA, USA) was cultured in Dulbecco-modified Eagle’s medium (DMEM, high glucose, Life Technologies, Warszawa, Poland) with addition of 10% heat-inactivated fetal bovine serum (FBS, Life Technologies, Warszawa, Poland), penicillin (100 μg/mL), and streptomycin (100 μg/mL). Cells were cultured in an atmosphere supplemented with 5% CO_2_ at 37 °C.

Zika virus (ZIKV, strain H/PF/2013) was acquired from BEI Resources, Manassas, VA, USA. Virus stocks were prepared by infecting confluent Vero cells with the ZIKV at 400 TCID_50_/mL. After four days of infection, cells underwent three freeze-thaw cycles. Then, supernatants were collected, aliquoted and stored at −80 °C. The infectivity of generated stock was measured using Reed and Muench titration method [31]. Mock-infected control was prepared in parallel using non-infected cells.

### 2.2. XTT Assay

In order to measure cell viability, commercially available kit (XTT Cell Viability Assay kit, Biological Industries, Cromwell, CT, USA) was used. Cells were incubated with tested compounds at for 3 days at 37 °C. Then, medium was discarded, and 100 μL of fresh medium was overlaid on cells. Next, 50 µL of activated 2,3-bis-(2-methoxy-4-nitro-5-sulfenyl)-(2H)-tetrazolium-5-carboxanilide (XTT) solution was added to each well and plates were incubated at 37 °C for 2 h. The absorbance was evaluated using Spectra MAX 250 spectrophotometer (λ = 490 nm; Molecular Devices, San Jose, CA, USA). Data are shown as the ratio of signal from the tested sample and the control sample (solvent-treated cells) × 100%.

### 2.3. RNA Isolation and RT-qPCR

Viral RNA isolation was carried out using a commercially available kit (Viral DNA/RNA Isolation Kit, A&A Biotechnology, Gdańsk, Poland) according to manufacturer’s protocol. For copy number quantification, isolated RNA was subjected to reverse transcription (R.T.) and quantitative real-time PCR (RT-qPCR) reaction using the GoTaq^®^ Probe 1-Step RT-qPCR System kit (Promega, Madison, WI, USA). The supernatants were diluted 1000-fold before isolation to prevent charged polymers from adversely affecting the isolation process [32].

The RT-qPCR reaction was conducted in 10 μL, containing 1 × GoScript™ RT Mix for 1-Step RT-qPCR, 1 × GoTaq^®^ Probe qPCR Master Mix with dUTP, specific probe labeled with 6-carboxyfluorescein (FAM) and 6-carboxytetramethylrhodamine (TAMRA) (5′-FAM-CGGCATACAGCATCAGGTGCATAGGAG- TAMRA-3′, 300 nM), and starters (5′-TTGGTCATGATACTGCTGATTGC-3′ and 5′-CCTTCCACAAAGTCCCTATTGC-3′, 450 nM). The reaction was carried out in CFX96 Touch™ Real-197 Time PCR Detection System (Bio-Rad, Warszawa, Poland). The RT-qPCR program consisted of the following steps: 15 min at 45 °C (reverse transcription), 2 min at 95 °C, then 40 cycles of 15 s at 95 °C and 30 s at 60 °C. To enable the quantification of the number of vRNA copies in a sample, standards were subjected to RT-qPCR. Briefly, PCR product was amplified and cloned into pTZ57R/T (Thermo Fisher Scientific, Warszawa, Poland) plasmid using the InsTAclone PCR cloning kit (Thermo Fisher Scientific, Warszawa, Poland). Obtained vector was amplified and linearized using HindIII restriction enzyme. Linear product was purified with the GeneJET PCR purification kit (Thermo Fisher Scientific, Warszawa, Poland) according to the manufacturer’s instructions. The concentration was assessed using a NanoDrop™ 2000 spectrophotometer (Thermo Fisher Scientific, Warszawa, Poland). The number of DNA copies was assessed and samples were serially diluted and used as an input for real-time PCR.

### 2.4. Virus Inhibition Assay

Vero cells were infected with 100 µL ZIKV (2000 TCID_50_/mL) in the presence of different concentrations of studied polymers. At 2 h post-infection, cells were washed thrice with PBS, and then were incubated with polymers for 3 days at 37 °C. Supernatants were collected, and the number of ZIKV RNA copies was assessed using RT-qPCR.

### 2.5. In Vitro Mechanism of Action

To determine at which step the PAMPS-PAaU copolymers hampered the ZIKV replication, a set of assays was performed as described before [24] with some modifications. In brief:

Assay I, “virus inactivation assay”, verified the direct inactivation of the virus by the tested compounds. Virus stock was incubated with copolymers (25 µg/mL) for 1 h at room temperature with mixing. Then, mixtures were diluted with medium to dilute virus stock to the final concentration of 10,000 TCID_50_/mL and copolymers below their active concentration (<1 µg/mL). Cells were overlaid with 100 µL of prepared samples and incubated for 2 h at 37 °C. Cells were washed three time with PBS, and fresh media was applied. Infection was carried out for 12 h at 37 °C, and supernatants were subjected to RT-qPCR analyses.

Assay II, “cell protection assay”, examined if compounds interact with the host cell and protect it from the viral infection. In order to test that, Vero cells were incubated with 100 µL of copolymers (25 µg/mL) in growth media for 1 h at 37 °C. Next, cells were washed with PBS three times and later infected with ZIKV at 10,000 TCID_50_/mL in 100 µL for 2 h. Next, cells were washed thrice with PBS to remove any residual virus. Then, fresh media was added and infection was carried out for 12 h at 37 °C. After incubation, supernatants were collected for RT-qPCR analyses.

Assay III, “virus entry assay”, allowed examining if tested polymers blocked the early steps of virus infection. Cells were overlaid with 100 µL of copolymer–virus mixture (10,000 TCID_50_/mL, 25 µg/mL) and infected for 2 h at 4 °C to allow the virus attachment, but not internalization into the host cells. Next, cells were washed with PBS three times and 100 µL of fresh media was added. Cells were incubated for 12 h at 37 °C, and supernatants were collected for RT-qPCR analyses.

Assay IV, “virus replication, assembly, and egress assay”, examined whether compounds inhibited ZIKV replication after virus entry. To test that, cells were infected with ZIKV with the dose of 10,000 TCID_50_/mL in 100 µL and incubated for 2 h at 37 °C. After infection, cells were rinsed thrice with PBS to remove any residual virus. Then, 100 µL of tested copolymers in the growth medium was added at concentration of 25 µg/mL. Infection was carried out for 12 h at 37 °C, and supernatants were subjected to RT-qPCR analyses.

### 2.6. Fate of Nanoparticles after Interaction with Vero Cells

To answer the question regarding the fate of PAMPS-PAaU copolymer nanoparticles after incubation with Vero cells, two types of experiments were carried out. In the first one Vero cells were seeded on coverslips in 6-well plates and were incubated with 1000 µL of PAMPS_75_-*b*-PAaU_28_-*b*-F (25 µg/mL) for 1 h at 37 °C. Next, the medium was refreshed and after 0, 3, 6 and 12 h cells were fixed with 4% formaldehyde. Cells were washed thrice with PBS and nuclear DNA was stained using DAPI (0.1 μg/mL, Sigma-Aldrich, Poznan, Poland) for 20 min at room temperature. Cells were thoroughly washed with PBS and coverslips were mounted on glass slides and sealed for confocal imaging. 

In the second type of experiment, Vero cells were seeded on coverslips in 6-well plates and were incubated with 3000 µL of PAMPS_75_-*b*-PAaU_28_-*b*-F (25 µg/mL) for 1 h at 37 °C. The medium was refreshed, and Vero cells were cultured for 0, 1, 12 and 24 h. Then, cells were washed twice with PBS and 1 mL of methanol was added. An amount of 100 µL of each methanol extract was diluted and fluorescence spectra was collected using HITACHI F-7100 spectrofluorometer (excitation wavelength λ_ex_ = 450 nm). 

### 2.7. Fluorescence Microscopy

To evaluate the interaction of polymeric nanoparticles with the cells, Vero cells were seeded on coverslips in 6-well plates and were incubated with 1000 µL of PAMPS_75_-*b*-PAaU_28_-*b*-F (50 µg/mL) for 1 h at 37 °C. Cells were fixed using 4% formaldehyde, permeabilized with 0.1% Triton X-100 and washed with PBS thrice. Cells were immunostained with phalloidin conjugated with Alexa Fluor 647 (0.2 U/mL, Invitrogen, Warsaw, Poland) for 2 h. Next, cells were thoroughly washed with PBS and nuclear DNA was stained using DAPI (0.1 μg/mL, Sigma-Aldrich, Poznan, Poland) for 20 min at room temperature. Cells were washed with PBS, and coverslips with immunostained cells were mounted and sealed on glass slides. Images were acquired with an A1-Si Nikon (Tokyo, Japan) confocal laser scanning system coupled with Nikon inverted microscope Ti-E using a Plan Apo 100×/1.4 Oil DIC objective. Three-dimensional fluorescence images were generated using NIS-Elements AR 3.2 software (Nikon Europe BV, Amsterdam, The Netherlands). 

The experiment was carried out to monitor the interaction of ZIKV with cells in the presence or absence of polymers. Vero cells were seeded on coverslips mounted in 12-well plates (TPP, Trasadingen, Switzerland) to achieve 90% confluency. Then, cells were infected or mock-infected with 100 µL ZIKV (2000 TCID_50_/mL) in the presence or absence of different concentrations of studied polymers for 48 h. 

To study if polymer hampers ZIKV attachment to host cells, Vero cells were seeded on coverslips mounted in 12-well plates (TPP, Trasadingen, Switzerland) to achieve 90% confluency. Cell were pre-cooled to 4 °C and incubated with a mixture of virus stock and 50 µg/mL PAMPS_75_-b-PAaU_39_ in medium containing 0, 5 or 10% (250 µL) final concentration of FBS for 1 h at 4 °C. After incubation, cells were immediately fixed and immunostained. 

Cells were then fixed using 4% formaldehyde, permeabilized with 0.1% Triton X-100, and non-specific binding sites were blocked using 5% bovine serum albumin (BSA; Bioshop, Burlington, ON, Canada) in PBS for 2 h. Immunostaining was conducted using rabbit anti-ZIKV envelope antibody (GeneTex, Irvine, CA, USA) at concentration of 1 μg/mL for 2 h. Cells were washed thrice with PBS and incubated with 2.5 µg/mL of Atto 488 goat anti-rabbit secondary antibody (Sigma-Aldrich, Poznan, Poland) along with phalloidin conjugated with Alexa Fluor 647 (0.2 U/mL, Invitrogen, Warsaw, Poland) for 1 h. Next, cells were washed with PBS and nuclear DNA was stained using DAPI (0.1 μg/mL, Sigma–Aldrich, Poznan, Poland) for 20 min at room temperature. Cells washed with PBS, and coverslips with cells were mounted on glass slides in ProLong Diamond Antifade Mountant medium (Life Technologies, Eugene, OR, USA) and sealed. Fluorescent images were acquired using EVOS imaging system (Life Technologies, Carlsbad, CA, USA) and processed using ImageJ (version 1.52b) Fiji software (Madison, WI, USA) [33].

### 2.8. Statistics

The experiments were carried out in at least three replicates. The data are shown as means ± standard deviations (S.D.). The 50% inhibitory concentration (IC_50_) and 50% cytotoxic (CC_50_) values were assessed using the Graph Pad Prism 8.0. The selectivity index (SI) was calculated according to the formula SI = CC_50_/IC_50_.

The statistical significance of the data presented in the manuscript was assessed with non-parametric Kruskal–Wallis test. *p* values below 0.05 were considered significant. 

The log removal value (LRV) was calculated according to the formula LRV = log(C_i_/C_0_), where C_i_ is the number of viral RNA copies per milliliter in the sample from the culture treated with a given polymer and C_0_ is the number of viral RNA copies per milliliter in the control sample (untreated cells).

## 3. Results

### 3.1. Polymers

Homopolymers PAMPS_m_ (m = 40, 75 and 170) and a series of diblock PAMPS_75_-b-PAaU_n_ copolymers (*n* = 3, 12, 28, and 39, respectively) were synthesized via RAFT polymerization. The random copolymer P(AMPS_50_-co-AaU_50_) was obtained in free radical polymerization. The polymer compositions were confirmed by ^1^H NMR (Supplementary Materials, Figures S1–S3). The structures of these polymers are presented in Figure 1 and their characteristics are shown in Table 1. 

**Figure 2:**
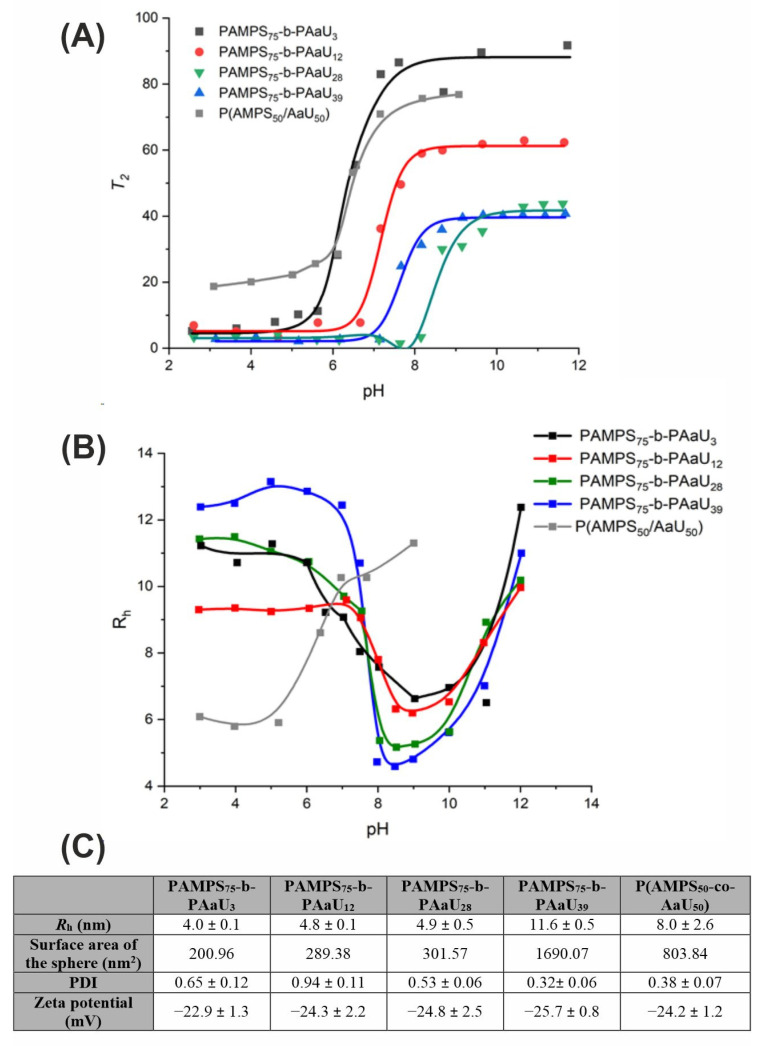
PAMPS-b-PAaU block copolymers form interpolymer micelles. Spin-spin relaxation time (*T*_2_) of ^1^H NMR signal at 1.25 ppm for PAMPS_75_-b-PAaU_3_ (black line), PAMPS_75_-b-PAaU_12_ (red line), PAMPS_75_-b-PAaU_28_ (green line), PAMPS_75_-b-PAaU_39_ (blue line) and P(AMPS_50_-co-AaU_50_) (grey line) as a function of pH in D_2_O containing 0.1 M NaCl at *C*_p_ = 10 g/L (**A**). Hydrodynamic radius (*R*_h_) for PAMPS_75_-*b*-PAaU_3_ (black line), PAMPS_75_-*b*-PAaU_12_ (red line), PAMPS_75_-*b*-PAaU_28_ (green line), PAMPS_75_-*b*-PAaU_39_ (blue line), and P(AMPS_50_-co-AaU_50_) (grey line) as a function of pH in 0.1 M NaCl aqueous solutions at *C*_p_ = 10 g/L and 25 °C (**B**). Values of hydrodynamic radius (*R*_h_), the surface area of the polymeric spherical nanoparticle, dispersity index (*Ɖ*) and zeta potentials for PAMPS-PAaU copolymers (*C*_p_ =1 mg/mL in PBS, pH= 7.4, T = 25 °C) (**C**).

All the polymers studied are well-soluble in water; however, due to their amphiphilic nature, they self-assemble in aqueous media with the formation of the core-shell micelles. The process is dependent on a pH of a solution and can be monitored by the analysis of the motion of polymer chains. ^1^H NMR spin-spin relaxation time (*T*_2_) was measured as a function of pH. Figure 2A shows *T*_2_ at 1.25 ppm, attributed to the pendant methylene protons in the PAaU block as a function of pH. At acidic conditions, *T*_2_ decreased due to the formation of a hydrophobic core of polymer micelles which restricted motions of the PAaU block.

These obtained data were verified using the dynamic light scattering (DLS) measurements of the dependence of hydrodynamic radius (*R*_h_) of macromolecules on pH (Figure 2B). The analysis of data presented in Figure 2A,B demonstrated that at acidic pH (pH~3), the polymers formed interpolymer micelles comprising PAaU core with PAMPS shells. When pH increased (pH~8), the polymers formed intrapolymer (unimer) micelles, i.e., each micelle was composed of a single polymer chain. The radius of intrapolymer micelles at pH = 7.4 was 8–11 nm (see Table S1) and reached a minimum value of about 5–7 nm for pH 8–9. Further increase in pH leads to stronger repulsive electrostatic forces between the pendant sulfonate ionic groups. At pH~12, the micelles completely dissociated, and polymeric chains expanded. The behavior of random P(AMPS_50_-co-AaU_50_) was, however, quite different. This polymer formed intrapolymer micelles even at acidic pH. With increasing pH, starting at about pH 5, the micelles began to uncoil, and the polymer chains adopted more and more extended conformation.

The values of *R*_h_ and zeta potential of PAMPS-b-PAaU micelles under the conditions relevant to the biological experiments (pH 7.4 PBS buffer, 37 °C) are presented in the table below (Figure 2C).

The results of the measurements presented above indicated that when considering the activity of PAMPS-PAaU copolymers in the biological experiments described below (pH~7.4), one should take into account that the macromolecules exist as negatively charged nanosized unimolecular micelles/nanoparticles with hydrodynamic radiuses being in the range 4–12 nm and zeta potentials around −25 mV (Figure 2A–C, Figures S4 and S5, Table S1). High values of zeta potential of the polymeric micelles provide stability of their nanodispersions in aqueous media. The comparison of the values of the surface area of the polymeric nanoparticles, calculated based on the assumption that they are ideally spherical, suggests that exposure/accessibility of the ionic sulfonate groups is expected to increase with the growing length of nonionic blocks which form the core of these micellar structures. 

### 3.2. Cytotoxicity and Cellular Localization of PAMPS-b-PAaU

**Figure 3:**
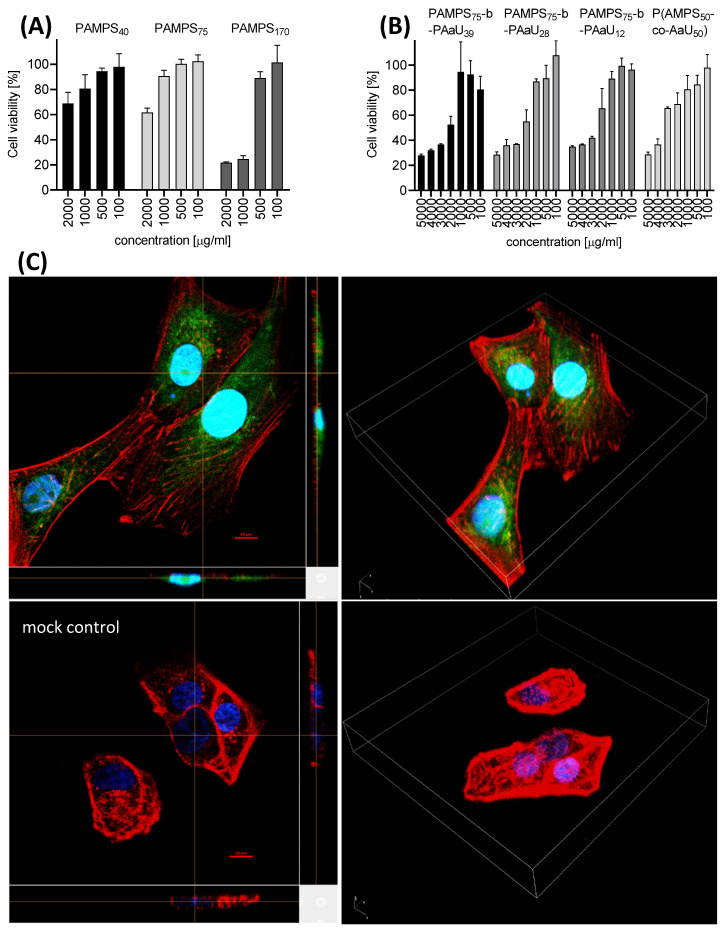
PAMPS-PAaU copolymers are not toxic towards Vero cells. Cytotoxicity of PAMPS_m_ homopolymers (**A**), PAMPS_75_-b-PAaU_n_ and P(AMPS_50_-co-AaU_50_) (**B**) copolymers of various M.W.s at 2000, 1000, 500 and 100 µg/mL. Results of XTT assay of the tested polymers on Vero cells. All experiments were performed in triplicate. Average values with standard error of the mean (error bars) are presented. (**C**) Three-dimensional fluorescent images of Vero cells incubated with 50 µg/mL of PAMPS_75_-*b*-PAaU_28_-*b*-F for 1 h. Cell nuclei are denoted in blue, actin is denoted in red and fluorescent polymer is green.

XTT assays were carried out to assess the cytotoxicity of tested copolymers towards cells. Vero cells were incubated with solutions of PAMPS_m_ homopolymers, PAMPS_75_-b-PAaU_n_ diblock copolymers and P(AMPS_50_-co-AaU_50_) random copolymer at various concentrations for 3 days (Figure 3A,B). Our results indicate low cytotoxicity of PAMPS polymers at concentrations up to 500 µg/mL. The introduction of PAaU block or random structure of PAMPS-PAaU further lowered the toxicity of studied copolymers, increasing CC_50_ > 2000 µg/mL. 

The interaction of studied copolymers with Vero cells was followed by observation of fluorescence laser scanning confocal images of the cells incubated with fluorescently labeled PAMPS_75_-*b*-PAaU_28_-*b*-F copolymer (Figure 3C and Figures S7–S10). Green fluorescence observed inside the cells indicated that the polymer nanoparticles localized in the cytoplasm shortly after addition. Further observations suggest, however, that they are eliminated from cells, because after 24 h after incubation only a very weak signal from PAMPS_75_-*b*-PAaU_28_-*b*-F was observed (Figure S10).

### 3.3. Antiviral Tests

**Figure 4:**
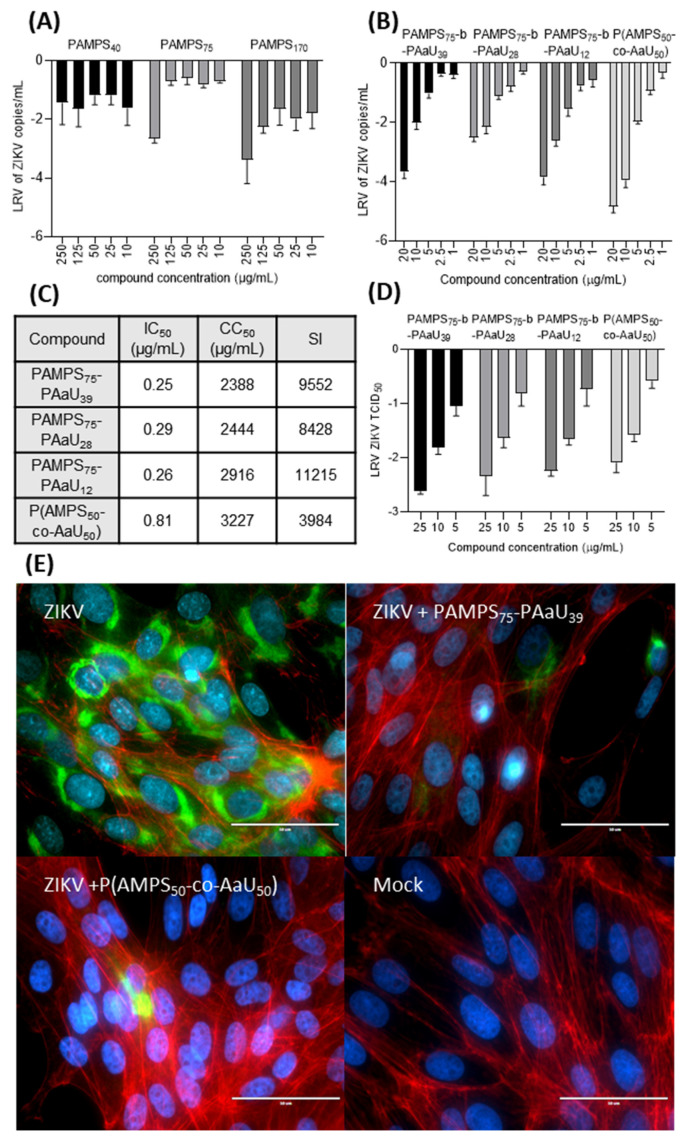
PAMPS_75_-b-PAaU_n_ block copolymers are potent inhibitors of Zika infection in vitro. (**A**) The inhibition of the ZIKV by PAMPS polymers. (**B**) PAMPS_75_-b-PAaU_n_ block copolymers and P(AMPS_50_-co-AaU_50_) copolymer at various concentrations in Vero cells. Inhibition of the infection was evaluated by RT-qPCR. Vero cells were infected in the presence of appropriate polymer concentrations for 3 days. Data are shown as logarithmic reduction values (LRV) of ZIKV RNA copy number per milliliter with SEM (error bars). (**C**) CC_50_, IC_50_ and selectivity index (SI) values for PAMPS-PAaU block and random copolymers. (**D**) Titration results of supernatants of Vero cells infected in the presence or absence of PAMPS_75_-b-PAaU_n_ block copolymers and P(AMPS_50_-co-AaU_50_) random copolymer in different concentrations. Data are shown as logarithmic reduction values of supernatant infectivity (TCID_50_) per milliliter of at least three replications. (**E**) Fluorescence images of cells infected with ZIKV (TCID_50_ = 2000/mL) in the presence or absence of the polymers at 25 µg/mL for 48 h. Cell nuclei are shown in blue, actin is shown in red and ZIKV E protein is denoted in green. Scale bar = 50 µm.

To assess the antiviral properties of studied macromolecules, Vero cells were infected with ZIKV in the presence or absence of tested polymers at different concentrations. Obtained data are summarized on Figure 4. PAMPS_m_ polymers showed weak potential to inhibit ZIKV, and only for PAMPS_m_ of higher M.W. a significant inhibitory effect at high concentrations was noted (250 µg/mL, Figure 4A). 

Surprisingly, adding a block of PAaU to PAMPS increased the inhibitory activity of the tested compounds. Block copolymers reduced the CPE at concentrations of 25 µg/mL or lower, and these observations were supported by the RT-qPCR data (Figure 4B and Figure S10). The strongest antiviral effect was shown for the polymers with the longest PAaU chain; however, the differences were slight. P(AMPS_50_-co-AaU_50_) showed similar antiviral properties, showing the highest reduction in ZIKV copy number at the highest concentrations, however, less inhibition at lower concentrations, resulting in a higher IC_50_ dose compared to PAMPS_75_-b-PAaU_n_ (Figure 4C). In contrast, PAMPS_75_-b-PAaU_3_ did not inhibit ZIKV replication in the tested concentration range (data not shown). This confirms that inhibitory properties of PAMPS_75_-b-PAaU_n_ correlate with the charge of polymer nanoparticles, which depends on the size of the PAaU block. The increase in the size of micelle core increases nanoparticle zeta potential, as more ionic groups are exposed at the particle surface in micelles with larger cores. To test this hypothesis, experiments using a series of PAMPS_75_-b-PAaU_n_ copolymers with longer PAaU blocks must be carried out. Higher antiviral activity of larger micelles is consistent with the assumption that the inhibitory mechanism involves the multivalent interactions of negatively charged ionic groups of polymers with positively charged glycoproteins located at the cell receptors.

Infectivity analyses of the supernatants from ZIKV samples treated with the tested copolymers confirmed the inhibitory effect (Figure 4D). All copolymers lowered the virus titers by ~2.5 orders of magnitude at 25 µg/mL in a dose-dependent manner. Fluorescence microscopy images also showed strong inhibition of ZIKV infection. While the majority of control cells showed ZIKV E protein production, only single cells were infected in the samples treated with the copolymers (Figure 4E).

### 3.4. Mechanism of Action

**Figure 5:**
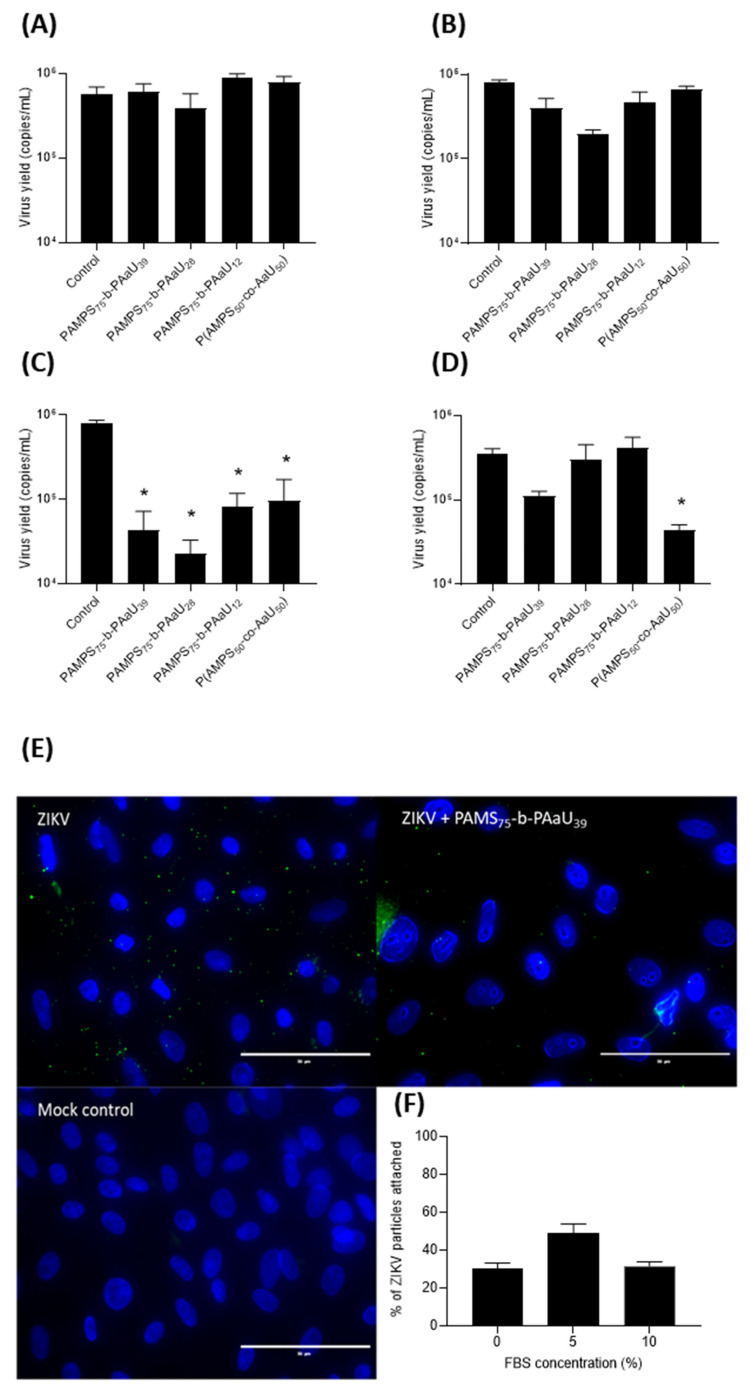
PAMPS_75_-b-PAaU_39_ inhibits the ZIKV replication cycle by blocking virus attachment. Inhibitory activity of copolymers was tested in Vero cells, as described in the Materials and Methods section: virus inactivation assay (**A**); cell protection assay (**B**); virus entry assay (**C**); virus replication, assembly, and egress assay (**D**). RT-qPCR was used to assess the number of viral RNA copies and to evaluate the inhibition of the infection. The experiments were carried out in triplicate. The data are shown as average values with SEM (error bars). * (*p* < 0.05) denotes values that are significantly different from the control. Representative fluorescent images of ZIKV particles on the cell surface (**E**). Vero cells were incubated with ZIKV in the presence or absence of PAMPS_75_-b-PAaU_39_ (25 µg/mL) at 4 °C. Cell nuclei are denoted in blue, and ZIKV E protein is denoted in green. The number of ZIKV virions attached to the cells (**F**). The individual counts, including the statistical error of the mean, are shown.

To investigate the mechanism of action for PAMPS-PAaU copolymers, a set of functional assays was carried out (Figure 5A–D). As shown in Figure 5A, the copolymers do not directly inactivate virions. After one hour of incubation with the copolymers and dilution of the copolymers, we observed no inhibition of infection, as was the case with preincubation of cells with compounds (Figure 5B). The results show that inhibition occurred early during the infection, but the polymers do not affect the virus itself (Figure 5C). This suggests that polymers can interfere with the process of virus attachment to the cell by the competition or changing the charge of the binding sites. To confirm these observations, cells were incubated with the virus in the presence or absence of copolymer at 4 °C to allow virus attachment to the cell surface, but not internalization. After incubation, samples were fixed and visualized by fluorescence microscopy (Figure 5E). An analysis of 10 random images of the control group and cells treated with the copolymer at a concentration of 25 µg/mL was performed. A significant reduction was found in the number of virus particles attached to the cell in the presence of PAMPS_75_-b-PAaU_n_ copolymers (Figure 5F). To test whether the serum could inactivate the copolymers, an attachment test was performed at different FBS concentrations. The results show no dependence of the blocking of ZIKV attachment to cells on the serum concentration. It is worth pointing out that our results also show a weaker inhibition at later steps of the virus replication cycle, especially in the case of P(AMPS_50_-co-AaU_50_) (Figure 5D). Therefore, an additional mode of action cannot be excluded, especially in light of the results showing that the copolymers penetrate the cell membrane and enter the cells. In conclusion, these results point out that PAMPS-PAaU copolymers inhibit the ZIKV replication cycle by blocking virus attachment to host cells.

## 4. Discussion

This work demonstrated that PAMPS-PAaU copolymers are efficient Zika virus inhibitors at submicromolar concentrations. The mechanistic studies indicated that they inhibit the ZIKV replication cycle by blocking virus attachment to host cells. The exact molecular mechanism of action remains to be fully understood. Based on the literature and PAMPS-PAaU composition, one can propose the involvement of several possible receptors. 

The rapid spread of the ZIKV in 2016 created high demand towards understanding the virus biology. The determination of the molecular mechanisms of early-stage ZIKV–host interaction and virus internalization would help in developing therapeutic strategies. However, the results are still not satisfactory. Research on skin-building cells has shown that human dermal fibroblasts, epidermal keratinocytes, and immature dendritic cells are permissive to ZIKV. Further research showed that ZIKV interacted with various types of non-specific and specific attachment factors. Like other flaviviruses, ZIKV uses heparan sulfate domains as non-specific attachment factors, assisting in virus interaction with primary receptors by retaining viral particles on the cell surface until they start to interact with the entry receptor. DC-SIGN and members of phosphatidylserine kinase family, AXL, Tyro3 and/or TIM-1 were identified as the specific ZIKV cellular attachment factors [34]. The same family of primary ZIKV receptors was proposed for other types of cells involved in virus transmission [35,36]. Recent proteomic studies allowed identification of the Neural Cell Adhesion Molecule (NCAM1) as a potential ZIKV receptor in Vero cells and human glioblastoma cells U-251 MG [37]. The findings mentioned above suggest that the attractive electrostatic interactions between negatively charged macromolecules of polymers used in current studies and positively charged domains of glycoproteins serving as the primary ZIKV attachment factors are essential for inhibition of the replication cycle. That is in agreement with earlier findings indicating the antiviral activity of heparin [38] and suramin [33]. The application of heparin as an antiviral agent is limited by its anticoagulant activity. Suramin, an approved antiparasitic drug, decreased the intercellular ZIKV RNA levels in a dose-dependent manner. Using the radioactive S-labeled ZIKV demonstrated that suramin interferes with virus attachment to the host Vero cells [35]. It was postulated that suramin binds to the (glyco)proteins of a ZIKV TIM1 receptor or ZIKV AXL receptor present on host cells, respectively [39]. Considering that in suramin and in the polymers used in our studies sulfonate groups are primarily involved in interaction with glycoproteins on a cell surface, one may assume that the same mechanism of action is involved.

Interestingly, we observed that the conformation of the polymeric chain strongly affects the efficiency of the virus inhibition. The macromolecules of PAMPS_75_-b-PAaU_n_ block copolymers forming negatively charged nanoparticles are much more efficient than PAMPS_75_ homopolymer, having the same number of AMPS units. That can be explained assuming that these polymeric nanoparticles can compete with virus particles for access to the attachment factor/primary cell receptor, and their size and charge density make it in favor of the polymer in this competition with the virus. As was demonstrated above, PAMPS-PAaU nanoparticles can finally enter the Vero cells tested, most likely by pinocytosis [40,41,42]. Although there is considerable literature on mechanisms of elimination of nanoparticles from the individual cells, more comprehensive studies are necessary to elucidate the exact mechanism. It was observed that nanoparticles could be exocytosed out of the cells, and the rate of the process was dependent on the chemistry of nanoparticles, and for a given type of nanoparticles, it was inversely proportional to their surface area [43,44]. 

Overall, our research shows a strong inhibitory effect of PAMPS_75_-b-PAaU_n_ copolymers on Zika virus multiplication in vitro. Copolymers show potential for further research into their antiviral properties. After a mosquito bite, Zika spreads throughout the human body through the blood and lymph vessels. The proposed copolymers could therefore act as intravenous drugs used in people at higher risk who have been diagnosed with Zika virus infection, especially in pregnant women. This is indicated by their advantageous physicochemical properties and high selectivity index (SI).

## 5. Conclusions

A series of anionic PAMPS homopolymers as well as amphiphilic block and random PAMPS-PAaU copolymers were synthesized, and their antiviral activity against Zika virus was demonstrated. The polymers are very well-soluble in water, and they self-assemble in an aqueous solution. Under the conditions relevant to the biological experiments (pH 7.4 PBS buffer, 25 °C) the macromolecules of PAMPS_75_-b-PAaU_n_ block copolymers exist as negatively charged (zeta potential about −25 mV) nanosized micelles (4–12 nm). They inhibit the ZIKV replication cycle by blocking virus attachment to the host cells. The process is highly effective. Considering the extremely low toxicity of the PAMPS-PAaU copolymers and their high SI values, these polymers are promising drug candidates against ZIKV.

## Figures and Tables

**Table 1:** Molecular weights and compositions of the polymers.

Polymer	*M*_n_^a^ (×10^4^)	*Ɖ* ^a^ (×10^4^)	*M*_w_/*M*_n_^a^	D.P. (AMPS) ^b^	D.P. (AaU) ^c^
PAMPS_40_	0.94	1.21	1.28	40	0
PAMPS_75_	1.72	2.18	1.26	75	0
PAMPS_170_	3.93	4.65	1.18	170	0
PAMPS_75_-b-PAaU_3_	1.85	2.61	1.41	75	3
PAMPS_75_-b-PAaU_12_	3.20	4.53	1.42	75	12
PAMPS_75_-b-PAaU_28_	5.05	6.72	1.33	75	28
PAMPS_75_-b-PAaU_39_	7.63	9.36	1.23	75	39
P(AMPS_50_-co-AaU_50_)	4.38 ^d^	9.90 ^d^	2.26 ^d^	50 ^e^	50 ^e^

^a^*M*_n_, number average molecular mass, *M*_w_, weight average molecular mass, *Ɖ*, dispersity index were determined by SEC in H_2_O/CH_3_CN (80/20, *v/v*) 0.1 M NaNO_3_ solution calibrated with poly(sodium styrenesulfonate) standards. ^b^ D.P., degree of polymerization of AMPS determined by SEC in H_2_O/CH_3_CN (80/20, *v/v*) 0.1 M NaNO_3_ solution calibrated with poly(sodium styrenesulfonate) standards. ^c^ D.P., degree of polymerization of AaU determined by ^1^H NMR in D_2_O. ^d^ Determined by SEC using a mixed solvent of water and DMF (50/50, *v/v*) containing 50 mM LiBr as the eluent. ^e^ % mol, determined from ^1^H NMR in D_2_O at 95 °C.

## Data Availability

Data is contained within the article and in Supplementary Material associated with the article.

## Supplementary Materials

The following are available online at https://www.mdpi.com/article/10.3390/pharmaceutics14020309/s1, Figure S1: ^1^H NMR spectrum for PAMPS_75_-b-PAaU_39_ at *C*_p_ = 10 g/L in D_2_O at pH 10. The DP of the PAaU block was estimated by comparing the intensity of signals *c* and *b*. Figure S2: DLS profiles for PAMPS-PAaU copolymers (*C*_p_ = 1 mg/mL in PBS, T = 37 °C). Figure S3: Normalized UV-vis absorption (solid line, in water at pH 10) and fluorescence spectra (dotted line, in water, λ_ex_ = 420 nm) of PAMPS_75_-b-PAaU_28_-b-F. Figure S4: 3D Fluorescent images of Vero cells incubated with 25 µg/mL of PAMPS_75_-*b*-PAaU_28_-*b*-F for 1 h and taken after selected period of time. Cell nuclei are denoted in blue and fluorescent polymer is green. Figure S5: Fluorescent images of Vero cells incubated with 25 µg/mL of PAMPS_75_-*b*-PAaU_28_-*b*-F for 1 h and taken after selected period of time. Cell nuclei are denoted in blue and fluorescent polymer is green (Experiment 1). Figure S6: Fluorescence spectra of methanol extract of Vero cells incubated with PAMPS_75_-*b*-PAaU_28_-*b*-F (25 µg/mL) for 1 h and collected after selected period of time (Experiment 2), Table S1: The values of hydrodynamic radius (*R*_h_) for PAMPS-PAaU copolymers (*C*_p_ = 10 mg/mL in 0.1 M NaCl, pH ≈ 7.5, T = 25°C).
