# Supplementary material for: Self-Organized Nanoparticles of Random and Block Copolymers of Sodium 2-(Acrylamido)-2-methyl-1-propanesulfonate and Sodium 11-(Acrylamido)undecanoate as Safe and Effective Zika Virus Inhibitors
Source: Pharmaceutics. 2022 Jan 27;14(2):309. doi: 10.3390/pharmaceutics14020309 (PMC8876367; doi:10.3390/pharmaceutics14020309)
Supplement: Supplementary file 1 [file pharmaceutics-14-00309-s001.zip › pharmaceutics-1502911-supplementary.pdf]

pharmaceutics

Supplementary Materials: Self-Organized Nanoparticles of Random and Block Copolymers of Sodium 2-(acrylamido)-2-methyl-1-propanesulfonate and Sodium 11-(acrylamido)undecanoate as Safe and Effective Zika Virus Inhibitors

Pawel Botwina, Magdalena Obłoza, Maria Zatorska-Płachta, Kamil Kamiński, Masanobu Mizusaki, Shin-Ichi Yusa, Krzysztof Szczubiałka, Krzysztof Pyrc and Maria Nowakowska

Materials and Methods

Reagents

4-Cyanopentanoic acid dithiobenzoate (95%, CTA: chain transfer agent) and fluorescein *o*-acrylate (95%) were purchased from Sigma-Aldrich (St. Louis, MO, USA) and used as receive. 11-Aminoundecanoic acid (AaU) were prepared in accordance with the literatures [1]. 2-(Acrylamido)-2-methyl propenesulfonic acid (95%, AMPS), acryloyl chloride (98%), ethanol (96%), and 4,4′-azobis(4-cyanopentanoic acid) (98%, V-501) from Fujifilm Wako Pure Chemical (Osaka, Japan) were used as received without further purification. 2,2′-Azobis(isobutyronitrile) (98%, AIBN) was purchased from Sigma-Aldrich (St. Louis, MO, USA) and recrystallized from methanol. Methanol was dried over 4 Å molecular sieved and distilled. Water was purified with a Millipore Milli-Q System.

Synthesis of Sodium 11-(Acrylamido)undecanoate (AaU)

Synthesis of AaU monomer was performed according to procedure reported before [1] . Briefly, Acryloyl chloride (56.7 g, 0.625 mol) was added on ice to 0.6 M NaOH aqueous solution (1.5 L) of 11-aminoundecanoic acid (40.3 g, 0.20 mol) within 30 min. After 3 h, the solution pH was change to pH 3 using 6 M hydrochloric acid. The resulting precipitate was filtered and later washed twice with water. The crude product was purified by recrystallization from a mixture of acetone and *n*-hexane (1/3, *v*/*v*) three times and dried at 50 °C under vacuum to give 15.4 g (30.4%) of 11-(acrylamido)undecanoic acid. AaU was prepared by neutralization of 11-(acrylamido)undecanoic acid (15.4 g, 60.7 mmol) with an equivalent of NaOH (2,43 g, 60.8 mmol) in methanol followed by precipitation of the salt with diethyl ether. The compound was dried at 50 °C under vacuum, and finally 16.1 g (95.7%) of AaU monomer was obtained.

Synthesis of $PAMPS_{40}$ and $PAMPS_{170}$

5,65 g of AMPS was dissolved in water and the resulting solution was neutralized with 1 M sodium hydroxide. Additional water (so that total solution volume was 5 mL) and 15 mg V-501 was added to the mixture. Then oxygen was removed from the solution by flushing argon through (bubbling) for 20 min. During this time, while stirring, the temperature of the mixture was raised to about 45 °C. Then 34.66 mg of CTA dissolved in 1 mL of ethanol was added dropwise to the mixture and the temperature was raised to 70 °C. For the next 7 ($PAMPS_{40}$) or 24 h ($PAMPS_{170}$), the mixture was stirred while maintaining a constant temperature and minimal argon flow. After this time, the post-reaction solution was dialyzed against water using a dialysis membrane with a molecular weight cut-off (MWCO) of 3 kDa. The purified polymer was isolated from the solution by lyophilization.

Synthesis of PAMPS Macro-Chain Transfer Agent (PAMPS Macro-CTA)

PAMPS Macro-CTA was obtained using the procedure described earlier [2]. AMPS (121 mmol, 25.0 g) was neutralized with NaOH (121 mmol, 4.81 g) in 60 mL of water, and CTA (0.829 mmol, 232 mg) and V-501 (0.166 mmol, 46.4 mg) were added to this solution. The mixture was deoxygenated with argon (30 min). Polymerization was carried out at 70 °C for 4 h. The polymer was dialyzed against pure water for seven days and recovered using a freeze-dry approach (conversion 83.9%, yield 23.2 g). The resulting PAMPS could be used as a macro-CTA.

Synthesis of PAMPS$_{75}$-b-PAaU$_{39}$

The synthesis was carried out using the procedure described earlier[3]. PAMPS macro-CTA (0.14 mmol, 2.43 g), AaU (7.43 mmol, 2.06 g), and V-501 (0.0165 mmol, 4.63 mg) were dissolved in water (13.5 mL). The solution was deoxygenated with argon (30 min). Block copolymerization was performed for 4 h at 70 °C. The diblock copolymer dialysed against a NaOH aqueous solution (1 × 10^{-6} M) at pH 8 for seven days (alkaline aqueous solution was changed twice a day). The diblock copolymer (PAMPS$_{75}$-b-PAaU$_{39}$) was recovered by a freeze-drying technique (yield 3.52 g, conversion 89.4%). The number-average molecular weight (M_n) and molecular weight distribution (M_w/M_n) of the polymer was assessed using gel-permeation chromatography (GPC) .

Synthesis of Random copolymer P(AMPS$_{50}$/AaU$_{50}$)

Synthesis of random copolymer P(AMPS$_{50}$/AaU$_{50}$) was carried out using the procedure described earlier [2]. Briefly, copolymerization of AMPS and AaU was carried out by conventional free-radical polymerization in the presence of AIBN in methanol. A procedure for the copolymerization is as follows: AMPS (4.14 g, 20 mmol) was neutralized with NaOH (0.89 g, 22 mmol) in 80 mL of methanol, and 5.55 g (20 mmol) of AaU and 16 mg (0.1 mmol) of AIBN were added to this solution. The methanol solution was outgassed on a vacuum line by six freeze-pump-thaw cycles. Copolymerization was performed for 12 h at 60 °C. The resulting sample was mixed with a large excess of diethyl ether to precipitate the polymer. The copolymer was purified by reprecipitation from a methanol solution into excess ether and then dissolved in water. The solution was dialyzed against a dilute NaOH aqueous solution (pH 8) for a week. The copolymer was recovered by a freeze-drying technique. The copolymer composition was determined from the intensity ratio of ^{1}H NMR resonance bands associated with the methylene protons in the AMPS unit (3.32 ppm) and the methylene protons neighboring the amide bond in the AaU unit (2.83 ppm) in D_2O at 95 °C.

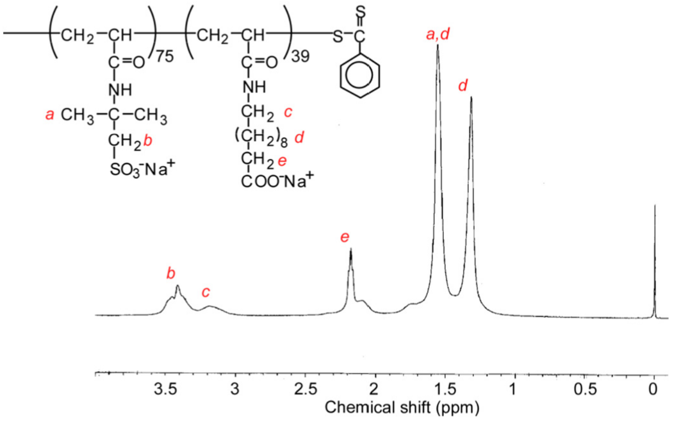

Figure S1. ^{1}H NMR spectrum for PAMPS$_{75}$-b-PAaU$_{39}$ at C_p = 10 g/L in D_2O at pH 10. The DP of the PAaU block was estimated by comparing the intensity of signals *c* and *b*.

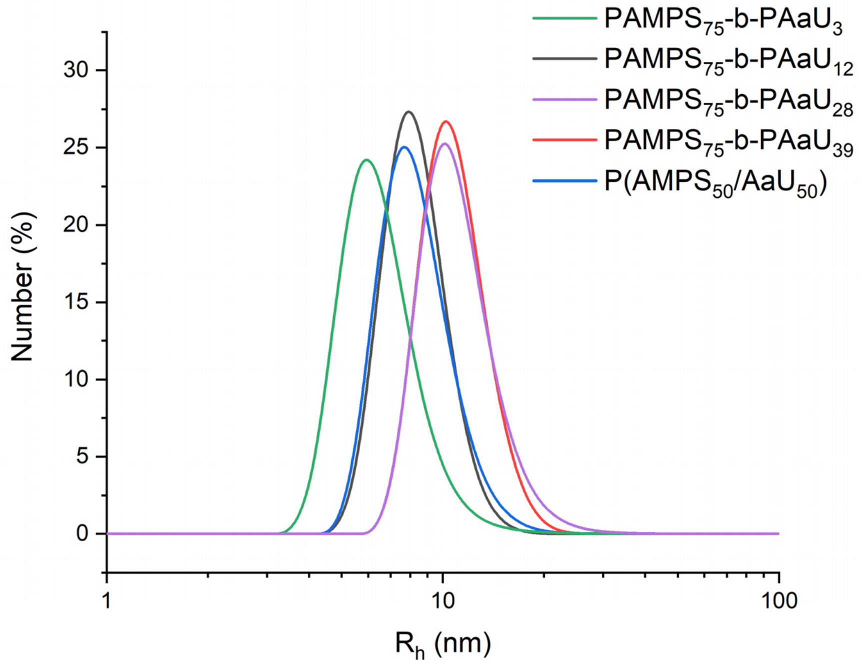

Figure S2. DLS profiles for PAMPS-PAaU copolymers (C_p =1 mg/mL in PBS, T = 37 °C).

Synthesis of $PAMSPS_{75}$-b-$PAaU_{28}$-b-F

$PAMPS_{75}$-b-$PAaU_{28}$ (M_n =5.05 × 10^4, M_w/M_n = 1.33, 25 mg, 5.0 × 10^{-6} mol), V-501 (0.3 mg, 1.0 × 10^{-6} mol), and fluorescein *o*-acrylate (19 mg, 5.0 × 10^{-5} mol) were dissolved in the mixture of MilliQ water and methanol (2:5, V = 1.75 mL). Argon was used for deoxygenation of the solution (for 20 min). Polymerization was performed at 70 °C for 1 h, and quenched by air. The fluorescent polymer was purified by dialysis against MilliQ water and recovered by a freeze-drying technique.

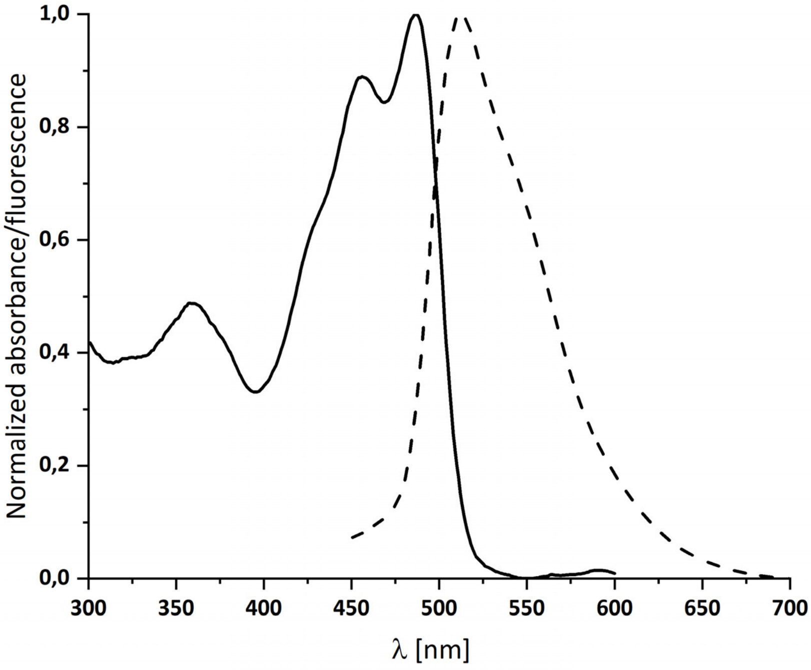

Figure S3. Normalized UV-vis absorption (solid line, in water at pH 10) and fluorescence spectra (dotted line, in water, λ_{ex} = 420 nm) of $PAMPS_{75}$-b-$PAaU_{28}$-b-F.

Table S1. The values of hydrodynamic radius (R_h) for PAMPS-PAaU copolymers (C_P = 10 mg/mL in 0.1 M NaCl, pH ≈ 7.5, T = 25 °C).

	**PAMPS$_{75}$-b-PAaU$_3$**	**PAMPS$_{75}$-b-PAaU$_{12}$**	**PAMPS$_{75}$-b-PAaU$_{28}$**	**PAMPS$_{75}$-b-PAaU$_{39}$**	**P(AMPS$_{50}$/AaU$_{50}$)**
**R_h [nm]**	8.04	9.06	9.26	10.70	10.27

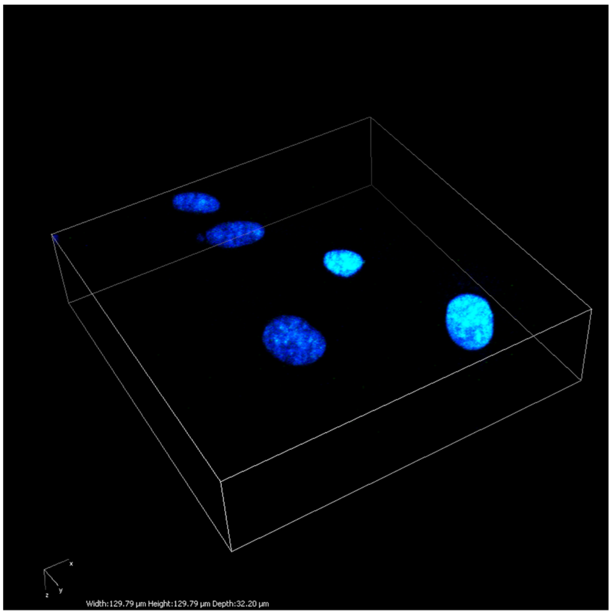

(**a**)

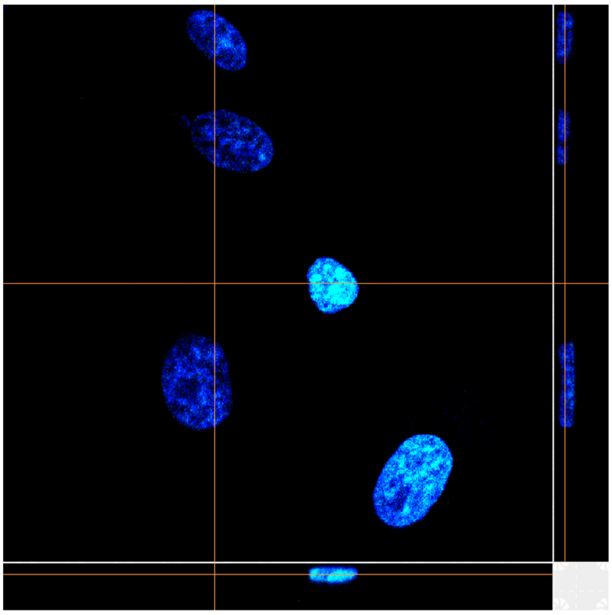

(**b**)

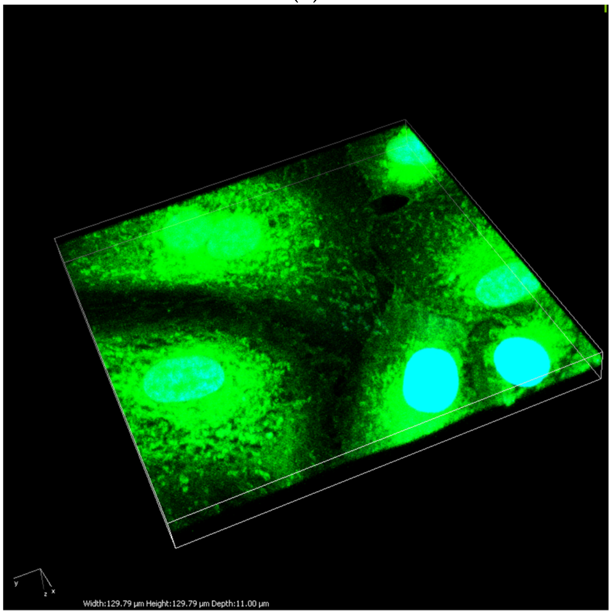

(**c**)

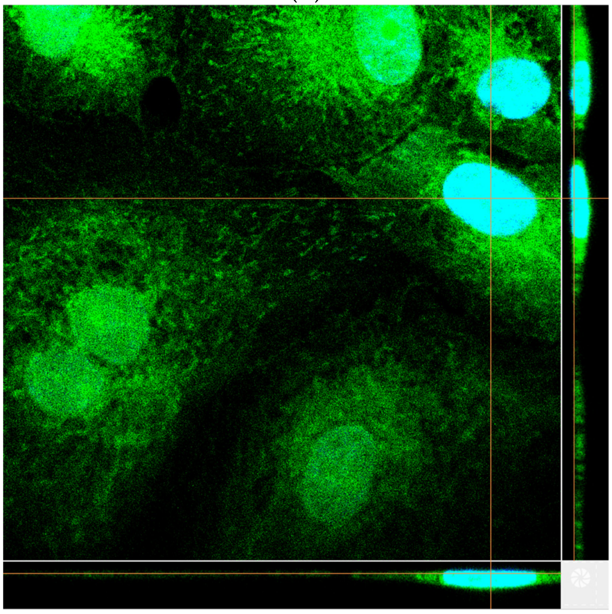

(**d**)

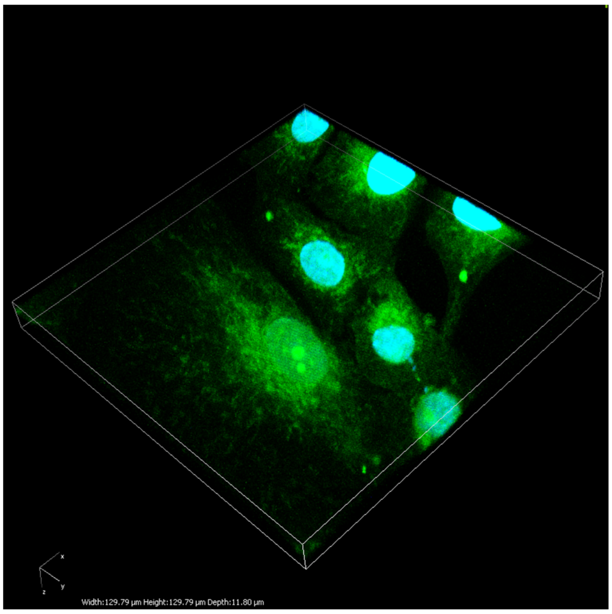
Width:129.79 µm Height:129.79 µm Depth:11.80 µm

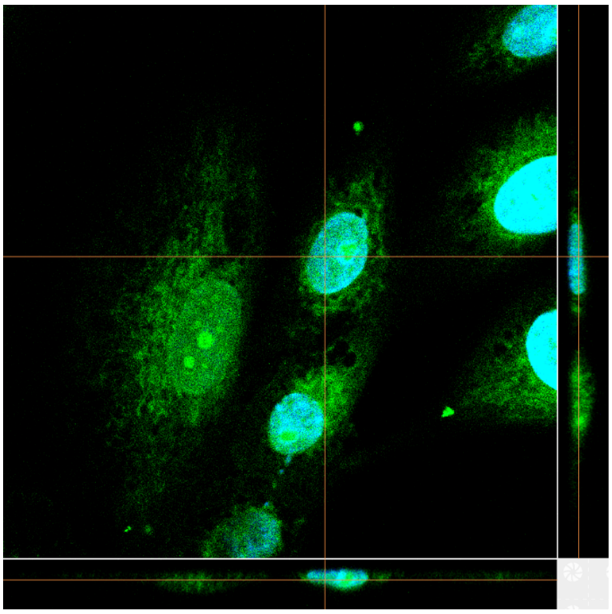

(**e**) (**f**)

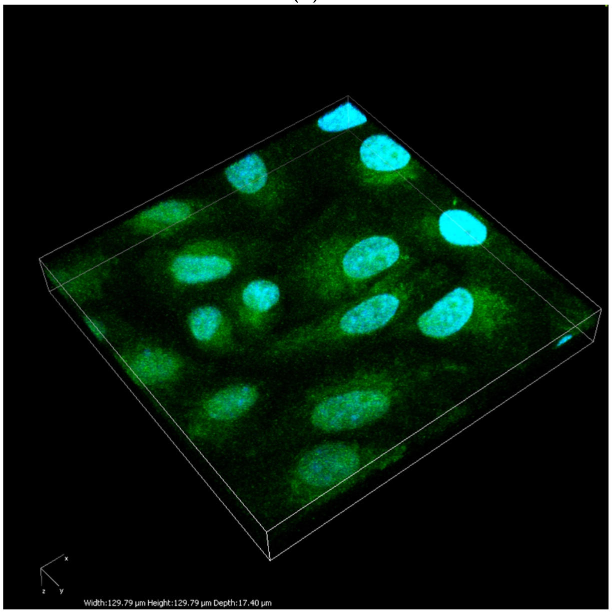
Width:129.79 µm Height:129.79 µm Depth:17.40 µm

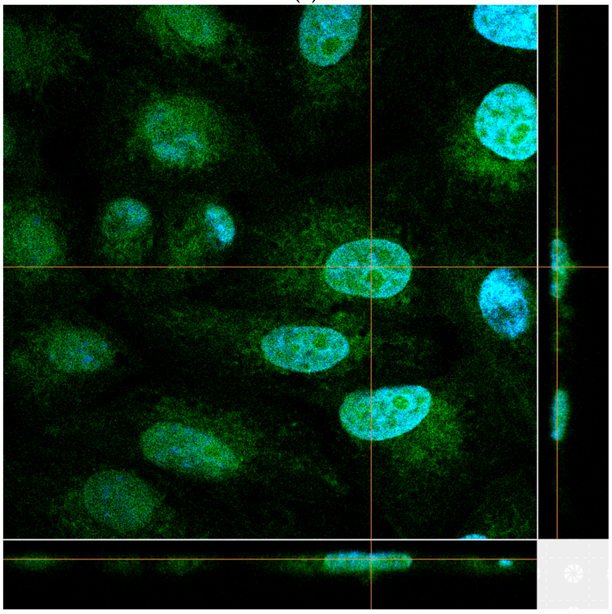

(**g**) (**h**)

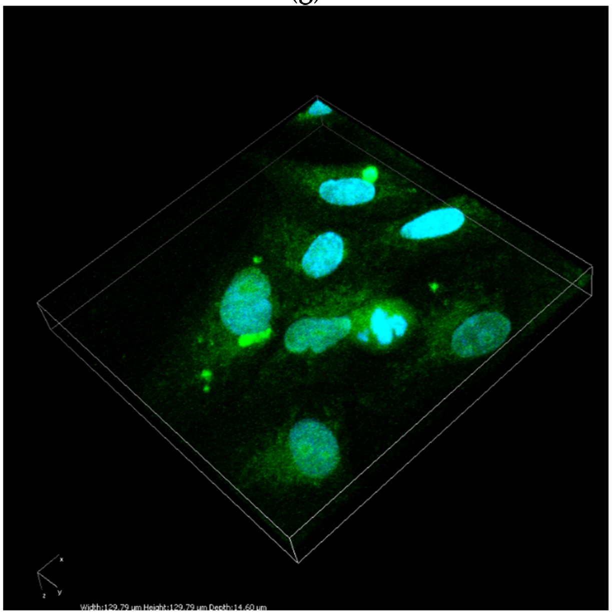
Width:129.79 µm Height:129.79 µm Depth:14.60 µm

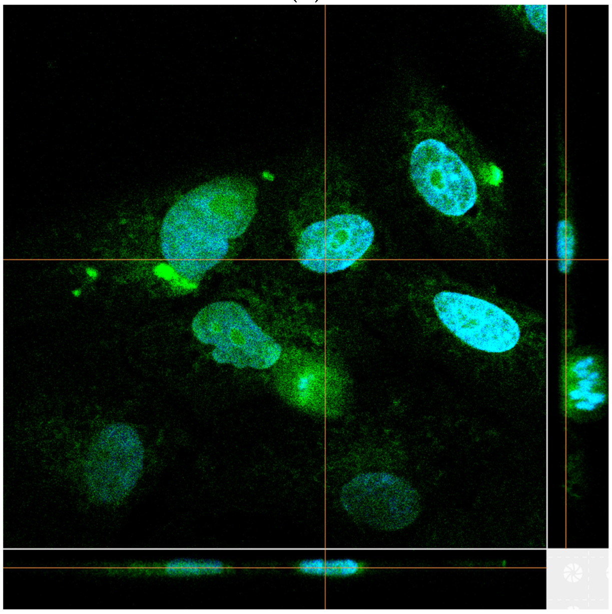

(i) (j)

Figure S4. 3D Fluorescent images of Vero cells incubated with 25 μg/mL of PAMPS$_{75}$-*b*-PAaU$_{28}$-*b*-F for 1 h and taken after selected period of time: (**a**,**b**) - mock control, (**c**,**d**)- 0 h, (**e**,**f**) - 3 h, (**g**,**h**) - 6 h, (**i**,**j**) - 12 h. Cell nuclei are denoted in blue and fluorescent polymer is green.

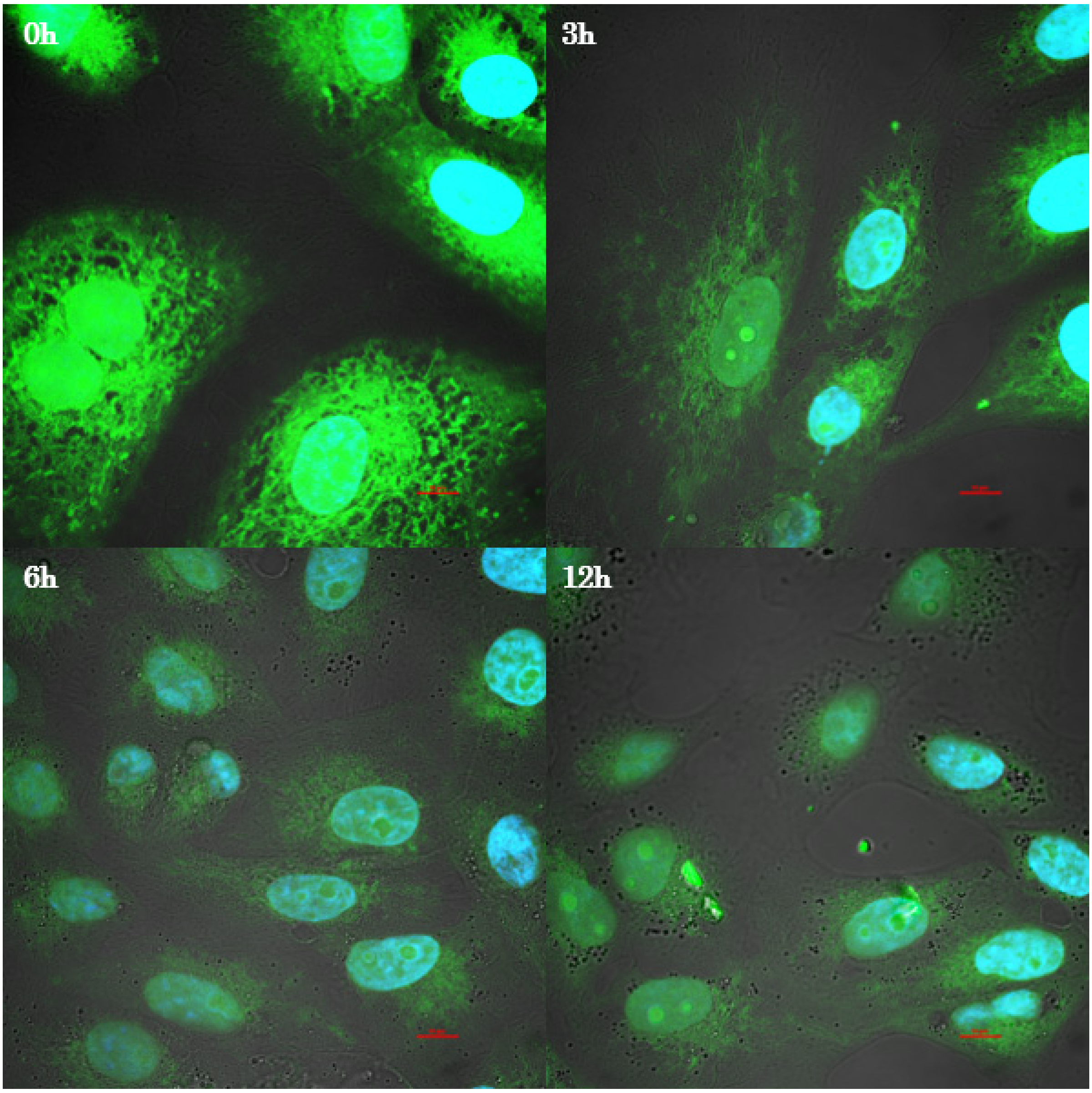

Figure S5. Fluorescent images of Vero cells incubated with 25 μg/mL of PAMPS$_{75}$-*b*-PAaU$_{28}$-*b*-F for 1 h and taken after selected period of time. Cell nuclei are denoted in blue and fluorescent polymer is green (Experiment 1). The scale bar corresponds to 10 μm.

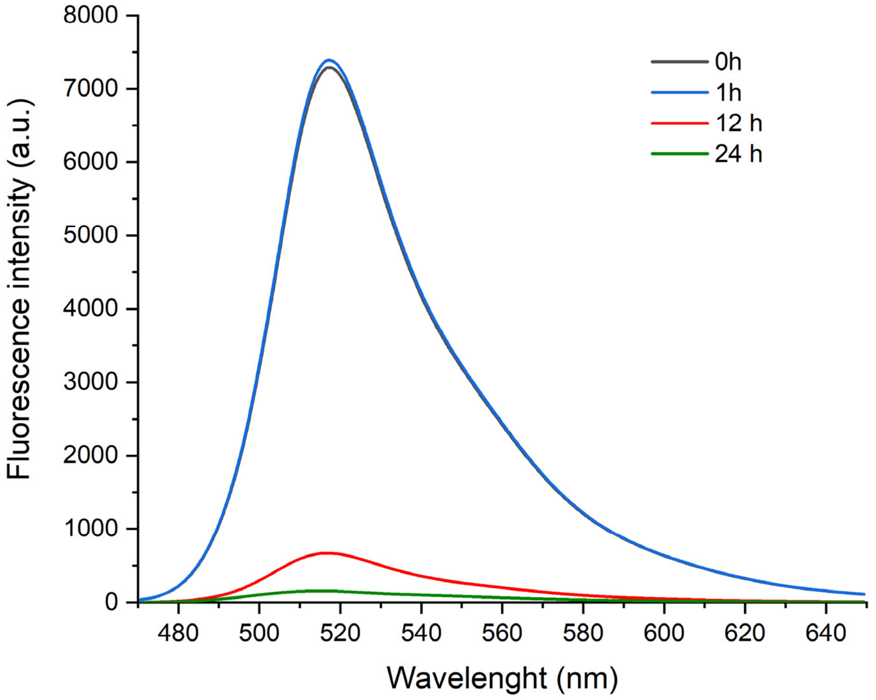

Figure S6. Fluorescence spectra of methanol extract of Vero cells incubated with PAMPS$_{75}$-*b*-PAaU$_{28}$-*b*-F (25 μg/ml) for 1 h and collected after selected period of time (Experiment 2).

References

1. Mizusaki, M.; Shimada, Y.; Morishima, Y.; Yusa, S.-I. pH-Responsive Intra- and Inter-Molecularly Micelle Formation of Anionic Diblock Copolymer in Water. *Polymers* **2016**, *8*, 56.
2. Yusa, S.-i., Sakakibara, A., Yamamoto,T., Morishima,Y., Reversible pH-induced formation and disruption of unimolecular micelles of an amphiphilic polyelectrolyte, *Macromolecules* **2002**, *35* (13), 5243–5249.
